# Differences in the respiratory response to temperature and hypoxia across four life-stages of the intertidal porcelain crab *Petrolisthes laevigatus*

**DOI:** 10.1007/s00227-018-3406-z

**Published:** 2018-08-23

**Authors:** Félix P. Leiva, Cristóbal Garcés, Wilco C. E. P. Verberk, Macarena Care, Kurt Paschke, Paulina Gebauer

**Affiliations:** 1 Centro i~mar, Universidad de Los Lagos, Casilla 557, Puerto Montt, Chile; 2 Department of Animal Ecology and Physiology, Institute for Water and Wetland Research, Radboud University, P.O. Box 9010, 6500 GL Nijmegen, The Netherlands; 3 Instituto de Acuicultura, Universidad Austral de Chile, Casilla 1327, Puerto Montt, Chile; 4 Centro FONDAP de Investigación en Dinámica de Ecosistemas Marinos de Altas Latitudes (IDEAL), Valdivia, Chile

## Abstract

For aquatic breathers, hypoxia and warming can act synergistically causing a mismatch between oxygen supply (reduced by hypoxia) and oxygen demand (increased by warming). The vulnerability of these species to such interactive effects may differ during ontogeny due to differing gas exchange systems. This study examines respiratory responses to temperature and hypoxia across four life-stages of the intertidal porcelain crab *Petrolisthes laevigatus*. Eggs, megalopae, juveniles and adults were exposed to combinations of temperatures from 6 to 18 °C and oxygen tensions from 2 to 21 kPa. Metabolic rates differed strongly across life-stages which could be partly attributed to differences in body mass. However, eggs exhibited significantly lower metabolic rates than predicted for their body mass. For the other three stages, metabolic rates scaled with a mass exponent of 0.89. Mass scaling exponents were similar across all temperatures, but were significantly influenced by oxygen tension (the highest at 9 and 14 kPa, and the lowest at 2 kPa). Respiratory responses across gradients of oxygen tension were used to calculate the response to hypoxia, whereby eggs, megalopae and juveniles responded as oxyconformers and adults as oxyregulators. The thermal sensitivity of the metabolic rates (*Q*_10_) were dependent on the oxygen tension in megalopae, and also on the interaction between oxygen tension and temperature intervals in adults. Our results thus provide evidence on how the oxygen tension can modulate the mass dependence of metabolic rates and demonstrate changes in respiratory control from eggs to adults. In light of our results indicating that adults show a good capacity for maintaining metabolism independent of oxygen tension, our study highlights the importance of assessing responses to multiple stressors across different life-stages to determine how vulnerability to warming and hypoxia changes during development.

**Electronic supplementary material:**

The online version of this article (10.1007/s00227-018-3406-z) contains supplementary material, which is available to authorized users.

## Introduction

Water temperature notably affects the balance between oxygen supply and demand in aquatic ectotherms (Verberk et al. 2011). Hence, an oxygen perspective may be useful to explain thermal responses in metabolism, body size and differences in species richness across thermal clines as well as the vulnerability of ectotherms to global warming (Van Dijk et al. 1999; Verberk et al. 2011; Verberk and Bilton 2013; Horne et al. 2015). Thermal effects are largely inescapable for aquatic ectotherms, because the thermal conductivity of water is high and physiological processes at all levels of biological organization are impacted by temperature (Hochachka and Somero 2002; Tattersall et al. 2012). Temperature also strongly affects fitness traits (growth, locomotion, reproduction). The interaction of temperature with biological traits such as body mass or environmental stressors such as hypoxia may occur synergistically, limiting the performance of organisms and narrowing their window of thermal tolerance (Frederich and Pörtner 2000; Woods et al. 2009; Moran et al. 2010; Eliason et al. 2011; Verberk and Bilton 2013; Verberk et al. 2016b).

**Fig. 1:**
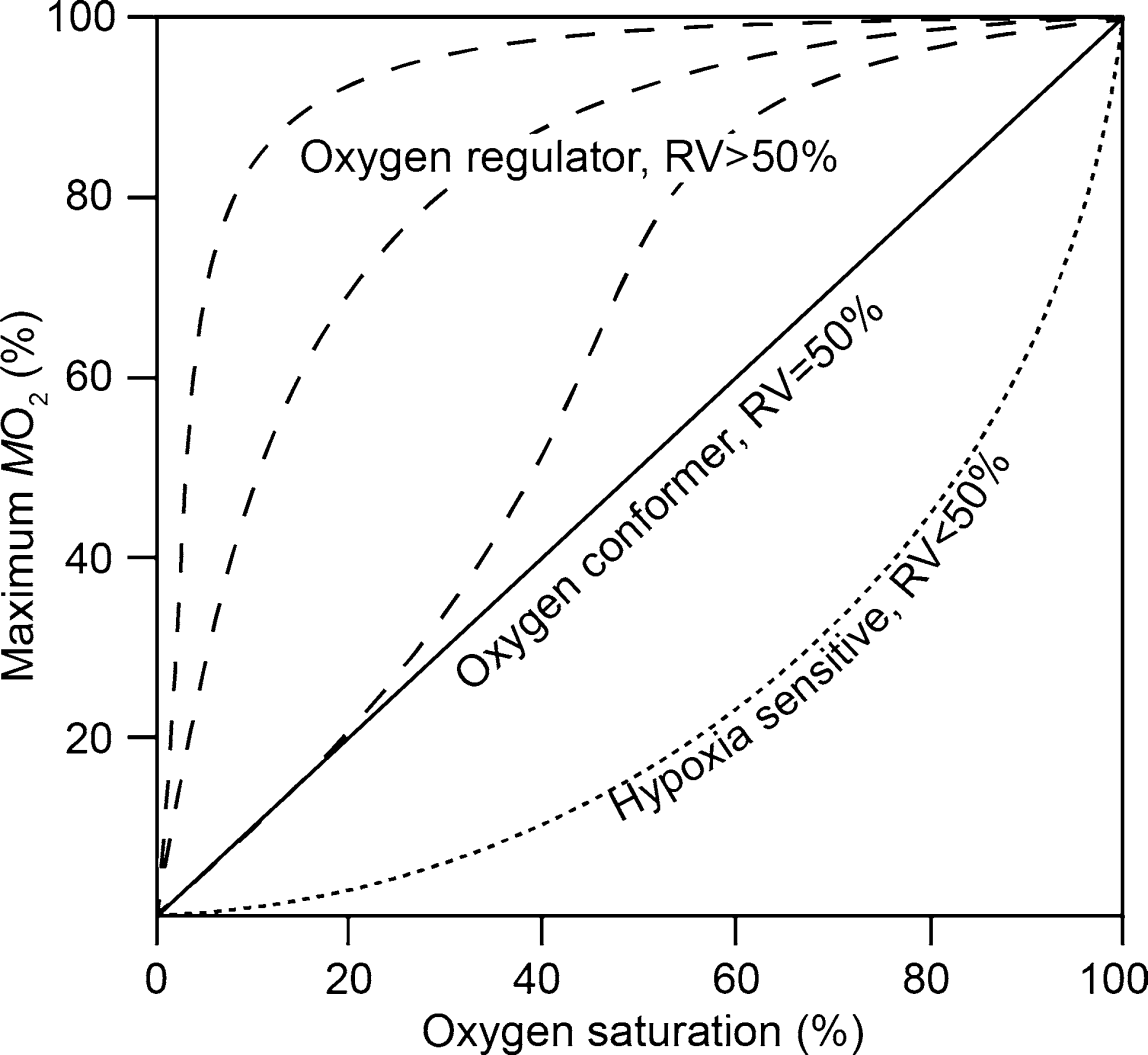
Schematic representation of hypothetical regulatory capacities across oxygen saturations and their associated oxygen regulation values (RVs, %). RVs are calculated using the area under each curve. An RV of 50% represents oxyconformers (solid line) and RVs above and below 50% denote oxyregulators (segmented lines) and hypoxia-sensitive individuals (dotted line), respectively. Modified from Alexander and McMahon (2004)

Animals can be classified as oxyregulators or oxyconformers depending on their respiratory response to hypoxia (Prosser 1955). Oxyregulators are able to maintain their oxygen consumption rates independently of ambient oxygen levels down to the so-called critical oxygen tension (*p*_crit_). Contrarily, the oxygen consumption of oxyconformers is largely dependent on ambient oxygen levels. Although the establishment of both categories has been subjected to extensive debate (van Winkle and Mangum 1975; Herreid 1980; Pörtner and Grieshaber 1993; Marshall et al. 2013) and the distinction is rarely absolute, a third and less explored response has been suggested. ‘Hypoxia sensitive’ describes the ability of certain organisms to rapidly decrease their metabolic rate upon slight decreases of oxygen tension (see Fig. 1). It is imperative to apply a quantitative method that covers these different responses (oxyregulators, oxyconformers and hypoxia sensitive) to provide a flexible representation of the inherent causes of variation in metabolic rates (Alexander and McMahon 2004; Mueller and Seymour 2011).

Studies on multiple stressors have shown that the early stages of marine invertebrates can be particularly susceptible to the effects of temperature and pH, with larvae being more sensitive than embryos (Przeslawski et al. 2015). Given that life-stages may differ in their vulnerability to multiple stressors, comparing changes in the physiological responses across different life-stages can help improve our understanding of the vulnerability of species to environmental challenges (Kroeker et al. 2013). Although previous studies have evaluated the effects of environmental stressors on the physiological characteristics of invertebrates, they are mostly focused on adult stages and frequently consider temperature as the main stressor. Here, we investigate how the interactive effect of temperature and oxygen tension can trigger different respiratory response patterns for a species of crustacean at different life-stages and modes of gas exchange. Decapod crustaceans are a group of invertebrates that mostly live in aquatic environments. They have complex life cycles, with contrasting physiological characteristics (e.g. related to oxygen uptake through either diffusion or convection) that differ not only across species, but also within species across different stages of development (Walther et al. 2009; Anger 2001; Storch et al. 2011; Jensen et al. 2013; Alter et al. 2015; Fitzgibbon et al. 2015). For eggs and probably larvae, oxygen required for metabolism is obtained primarily via diffusion. In contrast, gas exchange both in juveniles and adults occurs through convective processes, taking place primarily in the gills (Whiteley and Taylor 2015). Moreover, haemocyanin plays a more important role in carrying oxygen through an open circulatory system to the different tissues during these life-stages (Terwilliger 1998), especially at high temperatures (Giomi and Pörtner 2013). Given these morphological and functional differences across decapod life-stages, we hypothesize that responses at the whole-organism level to temperature and oxygen challenges should differ, resulting in a poor capacity to regulate metabolic rate in early life-stages (eggs and larvae) and better regulatory capacity in subsequent life-stages (juveniles and adults). To test this hypothesis, we measured metabolic rates in the benthic life-stages (eggs, megalopae, juveniles and adults) of the intertidal crab *Petrolisthes laevigatus* at three different temperatures and five different oxygen tensions in a fully factorial design (15 treatments per life-stage). This approach allowed us to estimate the effects of body mass on metabolic rate, compare the degree of respiratory control across life-stages and at different temperatures, and determine thermal sensitivity (*Q*_10_) across life-stages and at different oxygen tensions.

## Methods

### Animal collection and maintenance

Benthic life-stages of *Petrolisthes laevigatus* were collected in the intertidal zone (Pelluhuin) near Puerto Montt, Chile, between October 2009 (beginning of spring) and March 2010 (end of summer). Sea surface temperatures during the sampling period ranged from 12 °C (October 2009) to 15 °C (March 2010). Ovigerous females, newly settled megalopae, juveniles (carapace length, CL: 2–4 mm) and adults were transported to the Laboratory of Crustacean Ecophysiology (LECOFIC) at the Universidad Austral de Chile. Adults and juveniles were held in 16-L aquaria, megalopae in 0.8-L aquaria in a constant temperature room at 12 ± 1 °C, under a 12 h:12 h light/dark photoperiod without food. Both aquaria were supplied by an open-flow system of continuous filtered seawater (salinity 32, 12 °C). Only animals at the intermolt stage were used in the experiment. Eggs were obtained from ovigerous females using tweezers after 1 day in the laboratory. Eggs in the intermediate stage (between 25 and 50% of the yolk consumed with a barely visible ocular spot: sensu Lardies et al. 2004; Gebauer et al. 2007) from different females (*N* = 25, carapace length; CL: 12–13 mm) were used and pooled.

### Experimental setup

All life-stages were exposed to one of three temperature treatments (6, 12 and 18 °C) for 24 h, under normoxic conditions (21 kPa), and absence of food. These three temperatures fall within the range of spring and summer temperatures at the study location (4–18 °C; Gebauer et al. 2007). We used a thermostatized bath to increase the seawater temperature to 18 °C and a fridge connected to a thermostat (Danfoss EKC102A) to decrease it to 6 °C. For the intermediate acclimation (12 °C), incubations were set up inside the same temperature- and light-controlled room used for the aforementioned maintenance conditions.

After 24 h of normoxia exposure with the corresponding temperatures, each life-stage was exposed to each of the five nominal oxygen tensions: 2.3, 4.7, 9.4, 14.1 and 21.2 kPa (referred hereafter as 2, 5, 9, 14 and 21 kPa). The different oxygen tensions were attained by bubbling nitrogen gas (N_2_) through the seawater in a 200-L capacity reservoir tank followed by a 10 min equilibration period prior to use. All experiments were conducted inside a temperature- and light-controlled room during daytime to prevent diurnal cycles influencing measurements of metabolic rate. A 12 h:12 h light/dark photoperiod and UV-sterilized and filtered (1 μm) seawater were applied during incubations.

### Oxygen consumption rates

Closed respirometry was used to determine oxygen consumption rates (MR) of eggs, megalopae, juveniles and adults for every temperature/oxygen tension combination (3 × 5 = 15 combinations). Measurements of oxygen tension were made using a needle-type oxygen optic fibre connected to a Microx TX3 AOT (PreSens, Germany), which was calibrated prior to the experiment using a two-point calibration in water (0 and 100% air saturation). Oxygen concentration was measured before and after an incubation period of 3 h for adults, juveniles and megalopae and of 5 h for eggs. Oxygen content never decreased below 80% of initial values following these incubation periods, to prevent potential influences of accumulating metabolites and overlap between the different oxygen tension treatments. Given the differences in the volumes of each developmental stage, we incubated different numbers of animals in different volumes. For adults and juveniles, we allocated one individual per 1- and 0.25-L chamber, respectively. For megalopae, five individuals were incubated per 10-mL plastic disposable syringe, while 70 eggs were incubated per 6-mL plastic syringe. We used ten replicates per life-stage for each combination of temperature and oxygen, and an additional three controls per combination without individuals to estimate and correct for potential bacterial respiration (background respiration). On average, background respiration was never more than 5% of measured respiration rates. To determine dry weights, samples were lyophilized (Savant Novalyphe NL150) for a minimum of 48 h and then weighed (Precisa 290 SCS, ± 0.01 mg). Dry mass (DM) ranged from 5.18 to 7.63 mg for pooled eggs (*N* = 70), from 0.49 to 0.91 mg for individual megalopae, from 18.65 to 217.12 mg for individual juveniles and from 545.00 to 1571.10 mg for individual adults.

### Calculation and data analyses

Our data analyses were based on versions of linear models. A preliminary analysis indicated that mass-specific metabolic rate varied significantly between life-stage, temperature, oxygen tension as well as all the interactions between two or three of these factors (Table S1, Supplementary Information). However, as stage and body size are highly correlated, this model did not account for potential differences in mass-specific metabolic rate; so, we performed additional analyses to determine the effect of body mass (DM, g), oxygen tension (kPa) and temperature (°C) on the metabolic rate of *P. laevigatus*. Metabolic rate (*M*O_2_, µmol O_2_ h^−1^ ind^−1^) and body mass (DM, g) data were firstly log-transformed (base 10) and fitted to a series of models. The most informative model was selected using the lowest Akaike’s information criterion (AIC) (Table S2, Supplementary Information). As these models indicated that the temperature × body mass interaction was non-significant (ANOVA, *F*(1,584) = 0.09, *P* = 0.761, *N* = 588, Table S2), we decided to predict metabolic scaling relationships at different oxygen tensions while setting temperature at an average of 12 °C (Table S2, Supplementary Information). Thus, mass-scaling relationships for each oxygen tension level (2, 5, 9, 14 and 21 kPa) were fitted using the power function *Y* = *aM*^*b*^ where *Y* is the log-transformed metabolic rate, *a* is the constant (intercept), *M* is the log-transformed body mass of each life-stage and *b* is the scaling exponent (slope) (Kleiber 1932; West et al. 1997).

Oxygen regulation values (RV, %) were estimated according to Alexander and McMahon (2004) who used the zebra mussel *Dreissena polymorpha* as a model species. We calculated this respiratory index for each life-stage and experimental temperature with modifications adopted by Leiva et al. (2015). Regardless of oxygen tension, we assigned the highest oxygen consumption rate the value of 100% and transformed the oxygen consumption rates at the other oxygen tensions as a percentage of this highest value. Therefore, we obtained five different data points, one for each oxygen tension, and these oxygen tensions were transformed to a percentage of oxygen saturation. A third-order polynomial model (chosen on the basis of *R*^2^) was fitted to these five points and the area under the curve was calculated by integrating this equation between 0 and 100% of oxygen saturation. The value thus obtained reflects the regulatory capacity of an animal along an oxygen gradient (see Fig. 1). Thus, an oxyconformer will exhibit a value of 50% or close to this, while values above 50% indicate oxyregulatory capacity (becoming maximal at 100%). Values below 50% indicate that animals are sensitive to hypoxia (Alexander and McMahon 2004).

For each life-stage and oxygen tension, the thermal sensitivity was determined using the van ’t Hoff equation (*Q*_10_) as follows:

$$ Q_{10} = \left( {\frac{{{\text{MR}}_{2} }}{{{\text{MR}}_{1} }}} \right)^{{\frac{10}{{T_{2} - T_{1} }}}} , $$

where MR_1_ and MR_2_ are metabolic rates at temperatures *T*_1_ and *T*_2_ (when *T*_1_ < *T*_2_). Our three acclimation temperatures gave three temperature intervals, resulting in 60 *Q*_10_ values.

We assessed the effects of temperature and life-stage on the regulation values (RVs) using analysis of variance applied to linear models. This was followed by a Tukey pairwise comparison. In addition, *t* tests were used to assess whether life-stages are oxyregulators, oxyconformers or hypoxia sensitive (i.e. by comparing the mean of their RV against the threshold value of 50%). For these analyses, temperature was included as a categorical variable in our model (see Table S3, Supplementary Information). Similarly, we also applied analysis of variance to assess the effects of oxygen tension, life-stage and temperature intervals (∆Temp) on the *Q*_10_ values. Univariate normality assumptions were evaluated graphically by comparing the theoretical and observed distributions of residuals using *Q*–*Q* plots (Venables and Ripley 2002) and by applying the Shapiro–Wilk test. Homoscedasticity assumptions were evaluated with Levene’s test (Levene 1960) applying a significance level of 0.05. Residuals of the models were Box–Cox transformed to correct for heteroscedasticity for the *Q*_10_ analyses only (Box and Cox 1964). All analyses and the drafting of figures were carried out using R Statistical Software (R Core Team 2012).

## Results

**Fig. 2:**
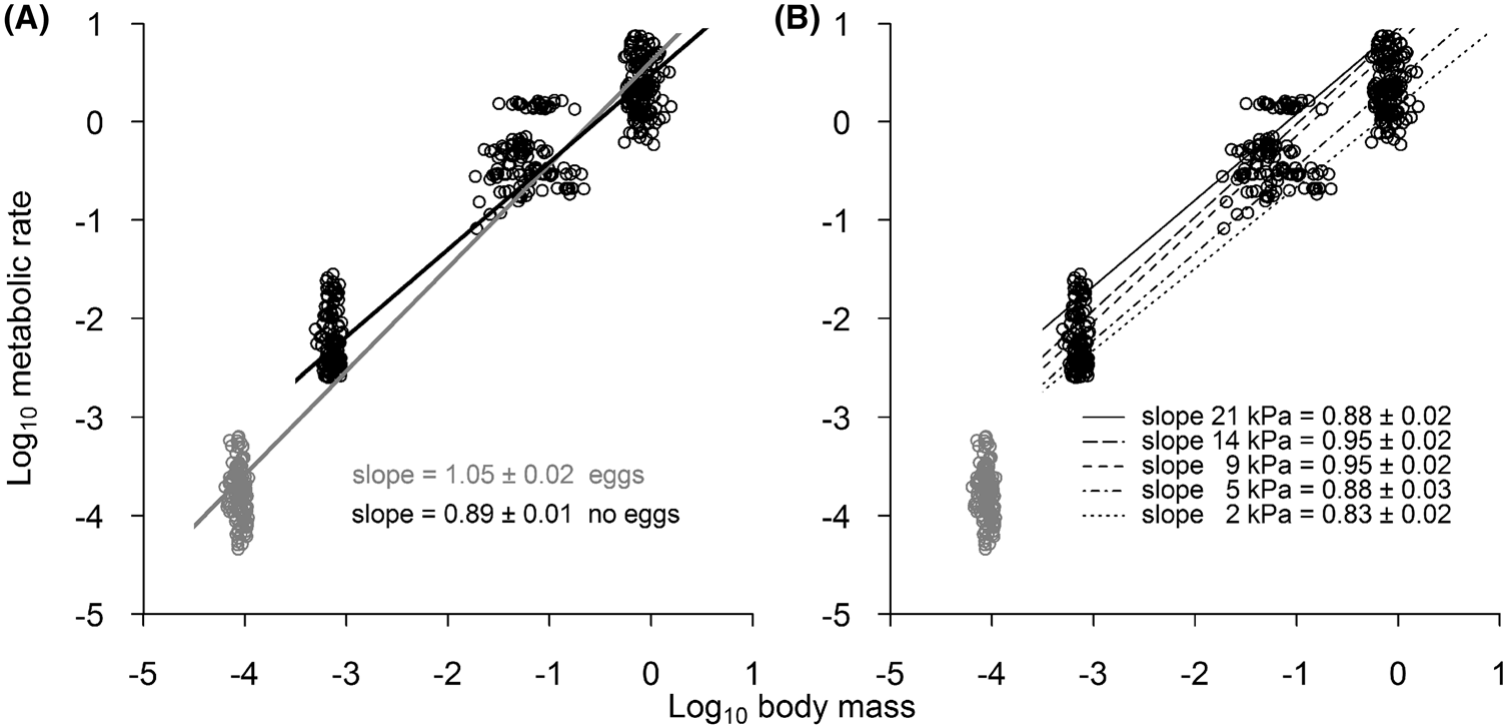
Mass scaling of the metabolic rate in different life-stages of *Petrolisthes laevigatus*. **a** Lines represent model fitted to data from all life-stages (solid grey line) and all life-stages except eggs (solid black line). **b** Lines represent model fits for each oxygen tension excluding eggs. All fitted lines represent the average of the three temperatures (12 °C). No significant interaction was found between temperature and body mass

Log-transformed metabolic rates were strongly related to the log-transformed body mass of *Petrolisthes laevigatus* scaling positively with an overall exponent of 1.05 ± 0.02, i.e. near isometric scaling (Fig. 2a). However, eggs demonstrated lower metabolic rates than expected for their body size. The model fit was greatly improved by accounting for this difference between eggs and other life-stages (i.e. by including a binary variable differentiating between eggs and non-eggs). This decreased the Akaike’s information criterion (AIC) value by 506.15 points. Metabolic rate scaled with body mass allometrically (0.89 ± 0.01) for the remaining three life-stages (Fig. 2a). Mass exponents also varied with oxygen tension (Log DM × oxygen tension: (ANOVA, *F*(4,576) = 9.22, *P* = 3.122e−07), reaching the lowest point (0.83 ± 0.02) at 2 kPa, and the highest point (0.95 ± 0.02) at 9 and 14 kPa (Fig. 2b and Table S2). Intermediate values of mass exponent were found at 5 and 21 kPa, being on average ca. 0.88 (Fig. 2b).

**Fig. 3:**
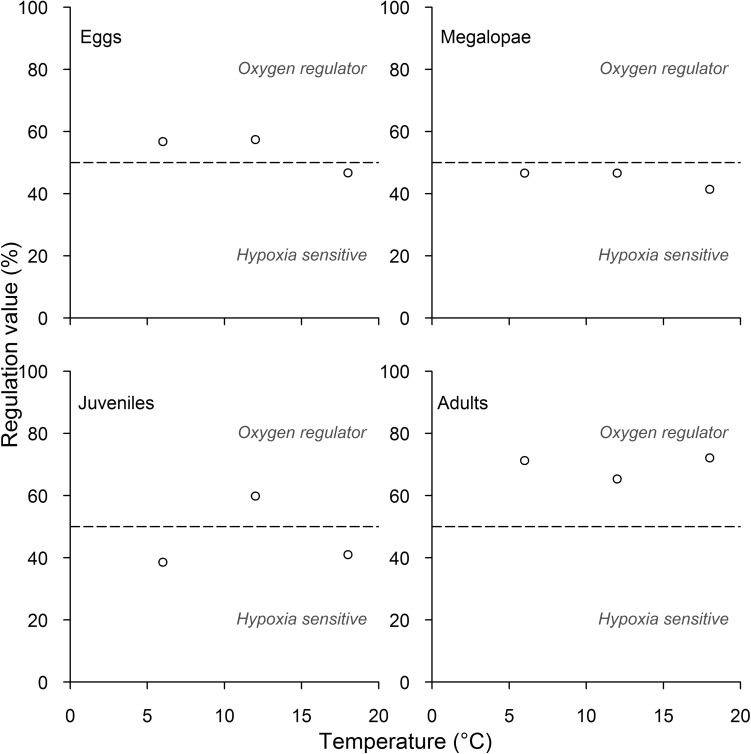
Oxygen regulation values (RVs, %) for each experimental temperature across all life-stages of *Petrolisthes laevigatus*. Oxyconformity is represented on each graph by a horizontal segmented line indicating an RV of 50%. Index values above or below this line represent oxyregulator or hypoxia-sensitive individuals, respectively

Regulation values (RVs, %) differed across *P. laevigatus* life-stages (ANOVA, *F*(3,6) = 7.90, *P* = 0.0166), but were not influenced by temperature (ANOVA, *F*(2,6) = 1.01, *P* = 0.4159) (Fig. 3 and Table 1). Our linear model indicated that the mean RV for eggs (53.61 ± 6.01%) was not significantly different from the mean RV for megalopae (44.89 ± 2.99%) or juveniles (44.46 ± 11.65%) (Tukey test, *P* > 0.05, Fig. 3). The average RV for the egg–megalopae–juveniles group was 47.65 ± 5.16% (not significantly different from 50%) and these life-stages were classified as oxyconformers (Fig. 3). In contrast, adults had a consistently higher RV (69.59 ± 3.69%) and were categorized as oxyregulators.

**Table 1:** Outcome of linear model using type II sums of squares showing the effects of temperature and life-stage on regulation values (RV) and oxygen tension, life-stage, and temperature intervals (∆Temp) on *Q*_10_ values of *Petrolisthes laevigatus*

Trait	Source	*df*	SS	*F*	*P* value
RV	Temperature	2	98.69	1.01	0.4159
	Stage	3	1147.91	7.90	**0.0166**
	Residuals	6	290.59		
*Q*_10_	Oxygen tension	1	0.44	3.37	0.0748
	Stage	3	0.90	2.29	0.0946
	∆Temp	2	0.31	1.18	0.3171
	Oxygen tension × stage	3	1.03	2.64	0.0645
	Oxygen tension × ∆Temp	2	0.73	2.80	0.0740
	Stage × ∆Temp	6	1.11	1.41	0.2353
	Oxygen tension × stage × ∆Temp	6	5.12	6.53	**0.0001**
	Residuals	35	4.57		

Bold indicates significant effects (*P* < 0.05)

Degrees of freedom (*df*), sum of squares (SS), Fisher (*F*) statistics and probability values (*P*) are indicated for each linear model

**Fig. 4:**
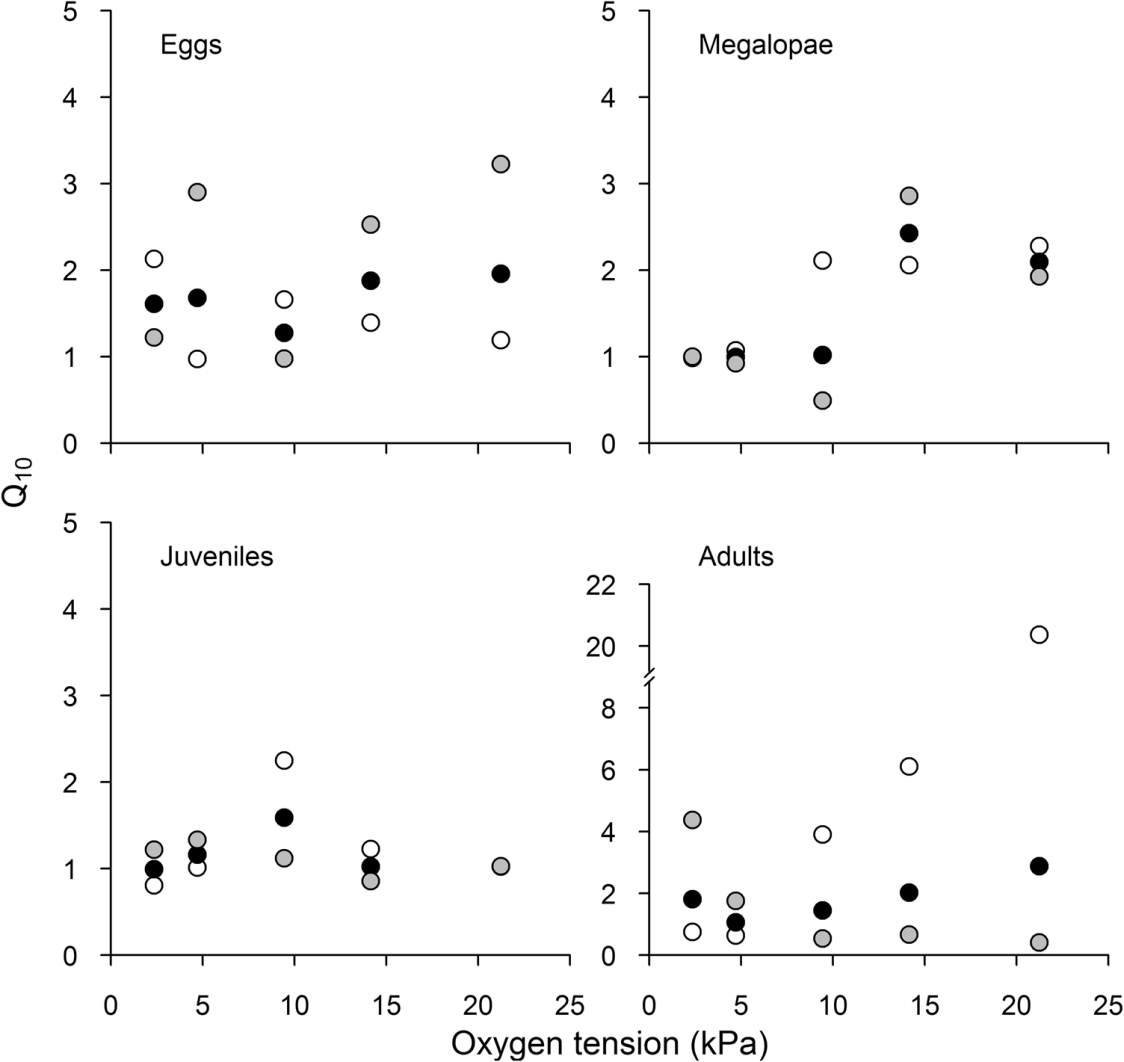
Thermal sensitivity of metabolic rate (expressed as a *Q*_10_ value) as a function of the oxygen tension (kPa) for different life-stages of *Petrolisthes laevigatus*. Black dots: 18 and 6 °C; grey dots: 18 and 12 °C; white dots: 12 and 6 °C. Note that *Q*_10_ values measured at 21 kPa overlap for juveniles

Thermal responses in oxygen consumption rates, measured as *Q*_10_ values, were affected by the interaction between oxygen tension, life-stage and ∆Temp (three-way ANOVA, *F*(6,35) = 6.53, *P* = 0.0001) (Fig. 4, Table 1 and Table S3 Supplementary Information). Simplified models of each life-stage showed that megalopae are affected by oxygen tension (two-way ANOVA, *F*(1,9) = 8.73, *P* = 0.0160) (Table 2) and that adults are affected by the interaction between oxygen tension × ∆Temp (two-way ANOVA, *F*(2,8) = 13.36, *P* = 0.0014) (Table 2). These results indicate that oxygen tension influences the ability of megalopae and adults to increase or decrease oxygen consumption in response to changes in environmental temperature.

**Table 2:** Outcome of linear model using type II sums of squares showing the effects of oxygen tension and temperature intervals (∆Temp) on *Q*_10_ values for each *Petrolisthes laevigatus* life-stage

Stage	Source	*df*	SS	*F*	*P* value
Eggs	Oxygen tension	1	0.071	0.63	0.4659
	∆Temp	2	0.182	0.81	0.4742
	Oxygen tension × ∆Temp	2	0.230	1.02	0.3980
	Residuals	9	1.014		
Megalopae	Oxygen tension	1	1.458	8.73	**0.0160**
	∆Temp	2	0.213	0.63	0.5499
	Oxygen tension × ∆Temp	2	0.005	0.01	0.9828
	Residuals	9	1.503		
Juveniles	Oxygen tension	1	0.008	0.10	0.7525
	∆Temp	2	0.009	0.06	0.9407
	Oxygen tension × ∆Temp	2	0.066	0.43	0.6624
	Residuals	9	0.698		
Adults	Oxygen tension	1	0.011	0.06	0.8045
	∆Temp	2	0.964	2.84	0.1166
	Oxygen tension × ∆Temp	2	5.552	13.36	**0.0014**
	Residuals	8	1.356		

Bold indicates significant effects (*P* < 0.05)

Degrees of freedom (*df*), sum of squares (SS), Fisher (*F*) statistics and probability values (*P*) are indicated for each linear model

## Discussion

Our approach provides metabolic rate estimations (ca. 600 measurements) in the intertidal crab *P. laevigatus* while exposed to different combinations of oxygen tension and temperature. These estimates allowed us to infer how biological (body mass and life-stages) and environmental (oxygen and temperature) modulators may affect the metabolism of this crustacean species.

We found that metabolic rate scaled allometrically with body mass in *P. laevigatus* across postembryonic life-stages. However, since mass-specific metabolic rates in eggs were lower than other life-stages, a lower mass exponent was calculated when only megalopae, juveniles and adults were considered (see Fig. 2a). The low metabolic rates observed in eggs are probably the result of yolk reserves that have been formed during the late stages of oogenesis (Nagaraju 2011). The yolk is metabolically inert, yet affects the body mass values and concomitantly results in lower mass-specific metabolic rates (Petersen and Anger 1997; Anger 2001). The oxygen demand of eggs increases as they develop and convert yolk into metabolically active tissue. At the same time, the gas exchange area remains relatively constant and the egg membrane acts as a diffusion barrier which can lead to a mismatch between oxygen supply and demand. The consequences of such oxygen limitation are hatching delays (Fernández et al. 2003) and subsequent catch-up growth even when favourable conditions for larval life are reinstated (Petersen and Anger 1997; Warkentin 2002; Horváthová et al. 2017). Several studies have shown changes in mass exponents through the ontogeny of different taxa which is in agreement with our findings (e.g. Killen et al. 2007; Frappell 2008). This includes crustacean studies. For example, in a study of the eastern lobster *Sagmariasus verreauxi*, the mass exponent changed from 0.97 in the planktonic phyllosoma stage to 0.83 in juveniles (Jensen et al. 2013). Glazier (2006) suggested that such transitions in the scaling from isometry to allometry are associated with an ontogenetic change in the surface area to volume ratio of respiratory organs. Such changes occur frequently among marine invertebrates with complex life cycles where the different life-stages exhibit large contrasts in morphology and physiology. In *P. laevigatus*, changes to the respiratory system occur during metamorphosis, where functional gills appearing in the juvenile stage become fully developed in adult life-stages. Although the presence of gills in zoea larvae has been suggested for other anomuran species such as *Lithodes santolla*, these do not apparently play a role in gas exchange (Paschke et al. 2010).

While studies that aim to evaluate the effects of environmental stressors (like temperature and oxygen tension) on the metabolic rate of crustaceans are common (e.g. Grieshaber et al. 1993; Burnett and Stickle 2001; Paschke et al. 2010; Leiva et al. 2015, 2016), only a few studies have evaluated whether these effects are differentially expressed for large and small bodied species (i.e. whether environmental variables modify the mass exponent). Studies on this topic have shown that temperature (Glazier 2005; Killen et al. 2010; Verberk and Atkinson 2013; Carey and Sigwart 2014) and oxygen tension (Urbina and Glover 2013) influence the mass exponent. Our study is one of the first that explores the effects of oxygen tension on the mass scaling of a crustacean species. In our study, we clearly demonstrate that oxygen tension alters the metabolic scaling in *P. laevigatus*; scaling exponents increase with increasing oxygen tension up to 9–14 kPa before declining again. Interestingly, Urbina and Glover (2013) showed that scaling exponents peaked at intermediate oxygen tensions in inanga *Galaxias maculatus* in a similar way. Although our limited data set does not allow for detailed inferences about the mechanistic basis of the observed response, it does show that physiological responses to low oxygen exposure co-vary with size and life-stage. Ontogeny-related processes such as the regulation of metabolic rates (Spicer and El-Gamal 1999), functional changes on subunits of oxygen transport proteins (Terwilliger and Brown 1993; Brown and Terwilliger 1999) and the development of the cardiovascular system (Harper and Reiber 2006; Rudin-Bitterli et al. 2016) could contribute to the variation in mass exponent in relation to oxygen tension described here. In our study, the largest life-stage (i.e. adults) had the strongest oxyregulatory capacity, and such physiological differences across *P. laevigatus* life-stages could explain the effect of oxygen tension on metabolic scaling: if under mild hypoxia, the oxygen consumption rates of the oxyconforming life-stages (i.e. the eggs, megalopa and juveniles) decreases, while the oxyregulatory adults are able to maintain oxygen consumption rates, this will result in a steeper scaling relationship at mild hypoxia, but not at normoxia or severe hypoxia (when the oxygen consumption rates of adults also decline). Interestingly, and in contrast to other studies (e.g. Killen et al. 2010; Carey and Sigwart 2014), any effect of temperature on metabolic scaling was evident.

All life-stages evaluated in this study spend most of their life span in the intertidal zone, usually on rocky shores. They are normally all exposed to thermal fluctuations in their habitats which explains why temperature effects in oxyregulatory capacity were similar at different life-stages. We predicted that variations in oxyregulatory capacity between life-stages also reflect the oxygen conditions found in their habitats. As expected, eggs’ metabolic rates decrease linearly with oxygen tension, probably as a result of their limited gas exchange system. This is perhaps not surprising, as the ventilation of eggs (and hence oxygen supply) is enhanced by the behaviour of ovigerous females, a form of parental care which reduces the need for active gas exchange. For example, an increase in oxygen supply as a result of abdominal flapping to eggs exposed to hypoxia of 2 kPa has been described for the hairy edible crab *Pseudograpsus setosus* (formerly *Cancer setosus*) (Fernández and Brante 2003).

Respiration patterns observed in this study are different from those recently described for the same species using the traditional (*p*_crit_) estimation (Alter et al. 2015). These authors found that eggs and juveniles were capable of maintaining their metabolism regardless of oxygen tension up to 15 and 5 kPa, respectively. For comparative purposes, we also estimated the *p*_crit_ according to Mueller and Seymour (2011) for all life-stages in our study. However, the absence of inflection points in the oxygen consumption of eggs, megalopae and juveniles prevented us from obtaining a reliable value for this estimator, restricting the results to the adult group (see Fig. S1, Supplementary Information). Differences in the origin of experimental animals may provide explanations for the inconsistencies between studies. To obtain eggs, Alter et al. (2015) reared their experimental ovigerous females in the laboratory, while megalopae were caught in the field and then reared until they metamorphosed to juveniles. In contrast, we obtained all life-stages from the field and maintained them for a short time in the laboratory. This suggests that environmental history may be important in shaping physiological performance to short-term exposures (Castillo and Helmuth 2005; Leiva et al. 2016). Future experiments should account for this initial variability. Moreover, future studies should determine whether later larval life-stages are more sensitive due to their larger mass and diffusion distances or because of their rudimentary cardiorespiratory anatomy, as suggested for other crustacean species (Fitzgibbon et al. 2015).

According to the oxygen and capacity-limited thermal tolerance (OCLTT) hypothesis (Pörtner 2001), a higher thermal sensitivity of oxygen demand should make an animal prone to heat stress because higher oxygen requirements imposed by warming cannot always be matched by a concomitant increase in oxygen supply. Conversely, a higher oxygen uptake capacity should make an animal less susceptible to heat stress. Indeed, some studies have found links between heat tolerance and both (1) thermal sensitivity of oxygen consumption rates (Verberk and Bilton 2011) and (2) differences in capacity for regulating oxygen uptake (Verberk and Bilton 2013). However, it is worth noting that high *Q*_10_ values can be interpreted both as having a high thermal sensitivity of oxygen demand or as having a high capacity for oxygen uptake (Verberk et al. 2016a). According to our results, *Q*_10_ values were dependent on the interaction between life-stage, oxygen tension and ΔTemp. The highest *Q*_10_ values were observed at higher oxygen tensions, especially in megalopae and adults. These values were statistically different from those observed during hypoxic conditions, suggesting that the newly settled megalopae used in our study show sensitivity to unstable environmental conditions similar to those present in the intertidal zone. Despite this, a high *Q*_10_ value was observed at 21 kPa for adults (ca. 20.37, Fig. 4) as a result of low metabolic rates observed at 6 °C (see Figs. S1 and S4, Supplementary Information). It remains unclear why these low metabolic rates occur.

In summary, our study demonstrates that environmental oxygen tension can affect the body mass scaling of metabolic rates in *P. laevigatus* and provides a good estimation of how respiratory capacity is depressed by oxygen supply. Such patterns demonstrate that different life-stages exhibit differences in oxyregulatory capacity. *P. laevigatus* adults represented the only life-stage that showed good capacity to maintain metabolism independent of oxygen tension. Other life-stages (eggs, megalopae and juveniles) were oxyconformers. These responses may reflect the environmental history of conditions experienced by these life-stages. Finally, our study adds evidence to the increasingly active debate on how different life-stages exhibit distinct responses to the effects of warming and hypoxia.

## Electronic supplementary material

Below is the link to the electronic supplementary material.

Supplementary material 1 (PDF 932 kb)
