# Supplementary material for: Differences in the respiratory response to temperature and hypoxia across four life-stages of the intertidal porcelain crab Petrolisthes laevigatus
Source: Mar Biol. 2018 Aug 23;165(9):146. doi: 10.1007/s00227-018-3406-z (PMC6132507; doi:10.1007/s00227-018-3406-z)
Supplement: Supplementary file 1 — Supplementary material 1 (PDF 932 kb) [file 227_2018_3406_MOESM1_ESM.pdf]

ELECTRONIC SUPPLEMENTARY MATERIAL

Differences in the respiratory response to temperature and hypoxia across four life-stages of the intertidal porcelain crab *Petrolisthes laevigatus*

Félix P. Leiva[1, 2, *], Cristóbal Garcés[1], Wilco C.E.P. Verberk[2], Macarena Care[1], Kurt Paschke[3, 4] and Paulina Gebauer[1]

[1] Centro i~mar, Universidad de Los Lagos, Casilla 557, Puerto Montt, Chile.

[2] Institute for Water and Wetland Research, Department of Animal Ecology and Physiology, Radboud University, P.O. Box 9010, 6500 GL Nijmegen, The Netherlands.

[3] Instituto de Acuicultura, Universidad Austral de Chile, Casilla 1327, Puerto Montt, Chile.

[4] Centro FONDAP de Investigación en Dinámica de Ecosistemas Marinos de Altas Latitudes (IDEAL), Chile.

Table S1 Outcome of linear model showing effects of different variation sources on metabolic rate of *Petrolisthes laevigatus*. Sum of squares (SS), Degrees of freedom (d.f.), Fisher (F) statistics, probability values (*P*), Akaike's Information Criterion (AIC) and adjusted R-squared (R^2) are indicated. Number of replicates: *N* = 7-10.

Source of variation	d.f.	SS	*F*	*P*-value	AIC	R^2
Stage	3	7529.3	291.55	< 2.2e-16	2952.241	0.8119
Temperature	1	1437.9	167.03	< 2.2e-16		
Oxygen tension	1	7130.8	828.38	< 2.2e-16		
Stage × Temperature	3	694.5	26.89	2.780e-16		
Stage × Oxygen tension	3	3680.9	142.53	< 2.2e-16		
Temperature × Oxygen tension	1	831.0	96.53	< 2.2e-16		
Stage × Temperature × Oxygen tension	3	635.8	24.62	5.383e-15		
Residuals	572	4923.8				

Table S2 Outcome of linear models showing effects of different variation sources on metabolic rate (LogMR) of *Petrolisthes laevigatus*. Sum of squares (SS), Degrees of freedom (d.f.), Fisher (F) statistics, probability values (*P*), Akaike's Information Criterion (AIC) and adjusted R-squared (R^2) are indicated for each linear model. Number of replicates: $N = 7\text{-}10$. Note that LogDM, oxygen tension and temperature were considered as continuous covariates, except for the last one where oxygen tension was categorical.

Model	Source of variation	d.f.	SS	*F*	*P*-value	AIC	R^2
~ LogDM	LogDM	1	1514.38	9453.3	<2.2e-16	595.8299	0.9415
	Residuals	586	93.87				
~LogDM × Temperature (see Fig. S2)	LogDM	1	1514.38	10074.81	<2.2e-16	560.379	0.9451
	Temperature	1	6.08	40.43	4.104e-10		
	LogDM × Temperature	1	0.01	0.09	0.761		
	Residuals	584	87.78				
~LogDM × Oxygen tension	LogDM	1	1514.38	12609.57	<2.2e-16	428.4211	0.9562
	Oxygen tension	1	23.31	194.10	<2.2e-16		
	LogDM × Oxygen tension	1	0.43	3.55	0.0599		
	Residuals	584	70.14				
~LogDM × Oxygen tension + Temperature (see Fig. 2A)	LogDM	1	1514.38	13871.83	<2.2e-16	373.3157	0.9602
	Oxygen tension	1	23.31	213.53	<2.2e-16		
	Temperature	1	6.46	59.21	6.077e-14		
	LogDM × Oxygen tension	1	0.45	4.15	0.04188		
	Residuals	583	63.65				
LogDM × Oxygen tension + Temperature + No eggs	LogDM	1	1514.38	32863.35	<2.2e-16	-132.8423	0.9832
	Oxygen tension	1	23.31	505.86	<2.2e-16		
	Temperature	1	6.46	140.27	<2.2e-16		
	No eggs	1	36.81	798.73	<2.2e-16		
	LogDM × Oxygen tension	1	0.47	10.28	0.001414		
	Residuals	582	26.82				
LogDM × Oxygen tension + Temperature + No eggs (see Fig. 2B)	LogDM	1	1514.38	34857.33	< 2.2e-16	**-161.5719**	0.9841
	Oxygen tension	4	24.19	139.22	< 2.2e-16		
	Temperature	1	6.45	148.35	< 2.2e-16		
	No eggs	1	36.61	842.62	< 2.2e-16		
	LogDM × Oxygen tension	4	1.60	9.22	3.122e-07		
	Residuals	576	25.02				

Table S3 Outcome of linear models showing effects of different variation sources on regulation values (RV, %) and thermal quotient (Q_{10}) of *Petrolisthes laevigatus*. Sum of squares (SS), Degrees of freedom (d.f.), Fisher (F) statistics, probability values (*P*), Akaike's Information Criterion (AIC) and adjusted R-squared (R^2) are indicated for each linear model. Most informative models are indicated by bold, based on lowest AIC values.

Model	Source	d.f.	SS	*F*	*P*-value	AIC	R^2
RV~ Temperature + Stage	Temperature (categorical)	2	98.69	1.01	0.41595	**86.2984**	0.6534
	Stage	3	1147.91	7.90	0.01661		
	Residuals	6	290.59				
	Temperature (numerical)	1	17.94	0.33	0.57917	87.2411	0.6204
	Stage	3	1147.91	7.21	0.01512		
	Residuals	7	371.34				
Q_{10}~ Oxygen tension × Stage	Oxygen tension (categorical)	4	0.82	0.81	0.5238	103.945	-0.0291
	Stage	3	0.89	1.17	0.3317		
	Oxygen tension × Stage	12	2.67	0.88	0.5720		
	Residuals	39	9.87				
	Oxygen tension (numerical)	1	0.38	1.68	0.2004	90.4771	0.0592
	Stage	3	0.89	1.28	0.2889		
	Oxygen tension × Stage	3	1.18	1.70	0.1778		
	Residuals	51	11.79				
Q_{10}~ Oxygen tension × Stage × ΔTemp	Oxygen tension (numerical)	1	0.44	3.37	0.0748	**66.5531**	0.4687
	Stage	3	0.90	2.29	0.0946		
	ΔTemp	2	0.31	1.18	0.3171		
	Oxygen tension × Stage	3	1.03	2.64	0.0645		
	Oxygen tension × ΔTemp	2	0.73	2.80	0.0740		
	Stage × ΔTemp	6	1.11	1.41	0.2353		
	Oxygen tension × Stage × ΔTemp	6	5.12	6.53	0.0001		
	Residuals	35	4.57				

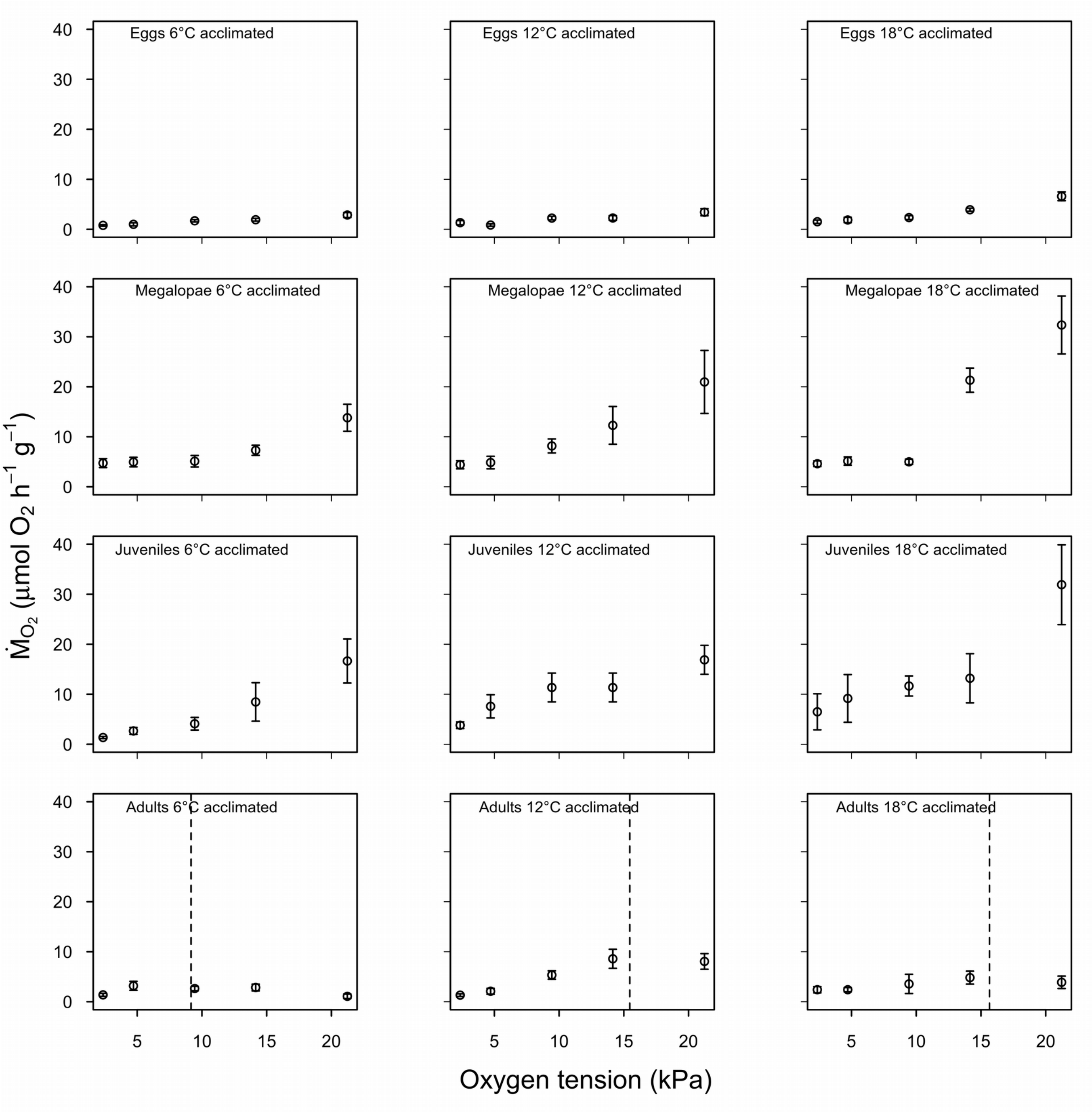

Fig. S1 Metabolic rates expressed as µmol of O_2 per hour per gram dry mass (M_{O2}, µmol O_2 h^{-1} g^{-1}) for each life-stage and temperature against oxygen tension (kPa). Values are expressed as means ± standard deviation. Vertical dashed lines in adults represents the critical oxygen tension (p_{crit}) calculated following to Mueller and Seymour (2011) with mean values for this index of 9.16, 15.46 and 15.76 kPa at 6°C, 12°C and 18°C, respectively.

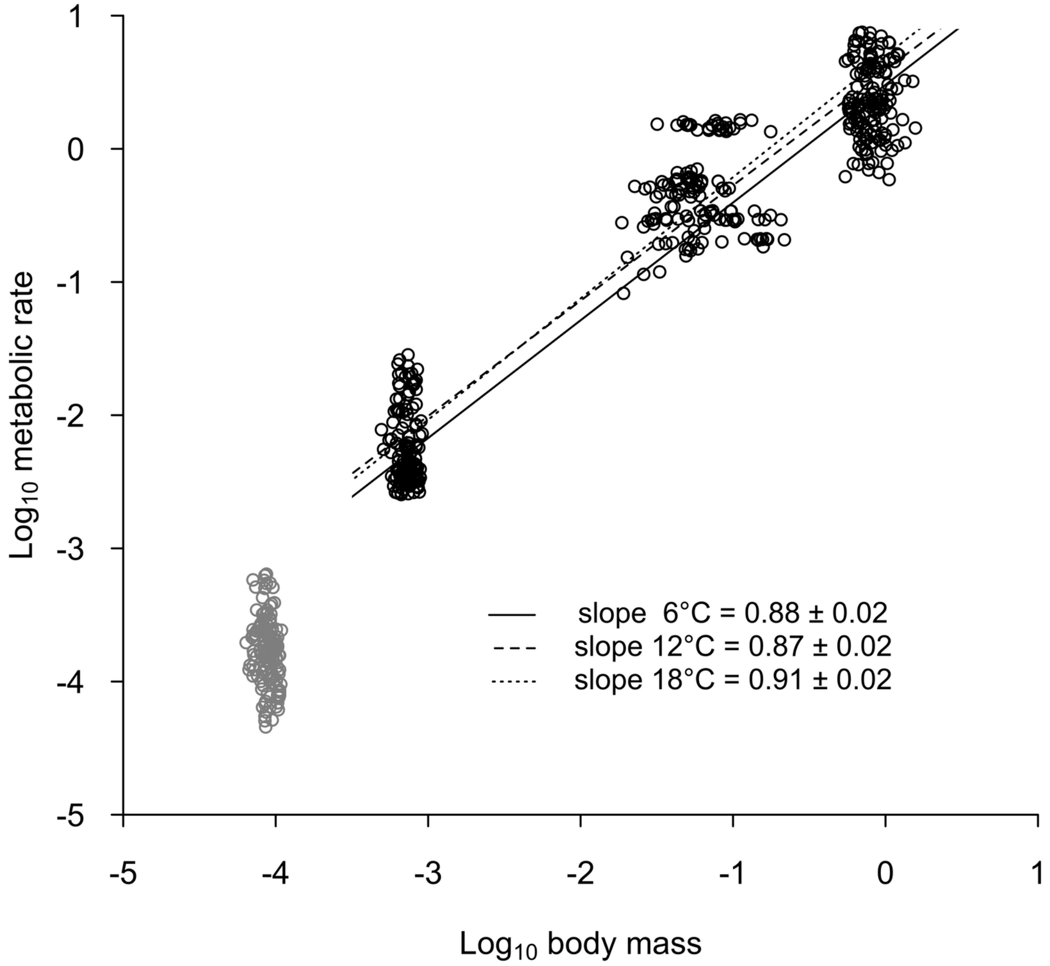

Fig. S2 Mass scaling for the log metabolic rate of *Petrolisthes laevigatus* for each temperature. Slopes were not different between 6°C, 12°C and 18°C (see Table S2, model 2) and these are only shown for illustrative purposes.

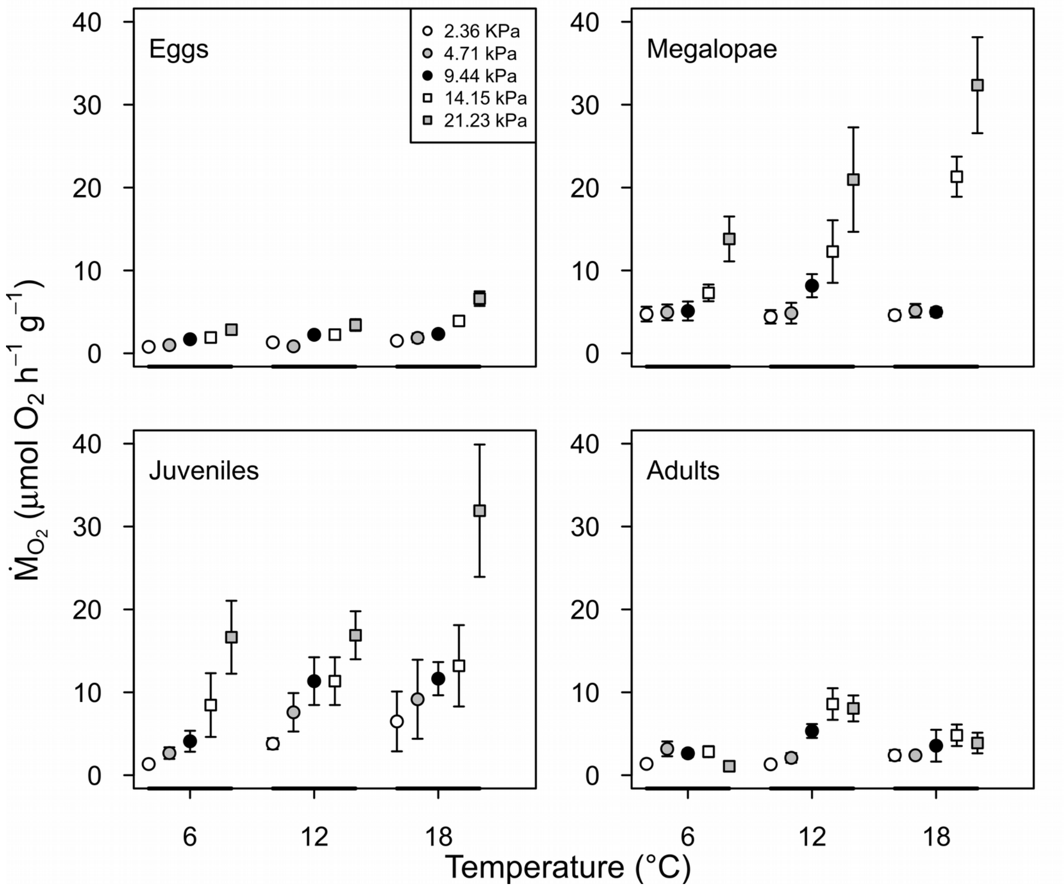

Fig. S3 Metabolic rate (log10-scale) of *Petrolisthes laevigatus* as function of temperature, for each life-stage. Dots with different colour or form represents each oxygen tension used during experiments (white circle: 2 kPa; grey circle: 5 kPa; black circle: 9 kPa; white square:14 kPa and black square: 21 kPa). Values are expressed as means ± standard deviation.
